# How do great bowerbirds construct perspective illusions?

**DOI:** 10.1098/rsos.160661

**Published:** 2017-01-18

**Authors:** Laura A. Kelley, John A. Endler

**Affiliations:** 1 Centre for Integrative Ecology, School of Life and Environmental Sciences, Deakin University, Waurn Ponds, Victoria 3216, Australia; 2 Department of Psychology, University of Cambridge, Downing Street, Cambridge CB2 3EB, UK; 3 School of Marine and Tropical Ecology, James Cook University, Townsville, Queensland 4811, Australia

**Keywords:** bowerbird, construction behaviour, forced perspective

## Abstract

Many animals build structures to provide shelter, avoid predation, attract mates or house offspring, but the behaviour and potential cognitive processes involved during building are poorly understood. Great bowerbird (*Ptilinorhynchus nuchalis*) males build and maintain display courts by placing tens to hundreds of objects in a positive size–distance gradient. The visual angles created by the gradient create a forced perspective illusion that females can use to choose a mate. Although the quality of illusion is consistent within males, it varies among males, which may reflect differences in how individuals reconstruct their courts. We moved all objects off display courts to determine how males reconstructed the visual illusion. We found that all individuals rapidly created the positive size–distance gradient required for forced perspective within the first 10 objects placed. Males began court reconstruction by placing objects in the centre of the court and then placing objects further out, a technique commonly used when humans lay mosaics. The number of objects present after 72 h was not related to mating success or the quality of the illusion, indicating that male skill at arranging objects rather than absolute number of objects appears to be important. We conclude that differences arise in the quality of forced perspective illusions despite males using the same technique to reconstruct their courts.

## 1. Introduction

Many animals build structures that are used to provide shelter, avoid predation, attract mates or house offspring [1]. Seemingly complex constructions can arise from relatively simple innate rules, such as the impressive multi-chambered nests built by termites [2], but some structures, such as elaborate bird nests, may involve learning and memory [3]. Constructions sometimes form part of courtship displays, and these extended phenotypes can provide unique information about male quality [4]. They may be used as a proxy for a male's cognitive ability, for example, their skill at construction or locating appropriate objects ([5,6], but see [7]). However, the behavioural and potential cognitive processes underlying the construction of non-bodily ornaments has rarely been investigated [8].

**Figure 1.**
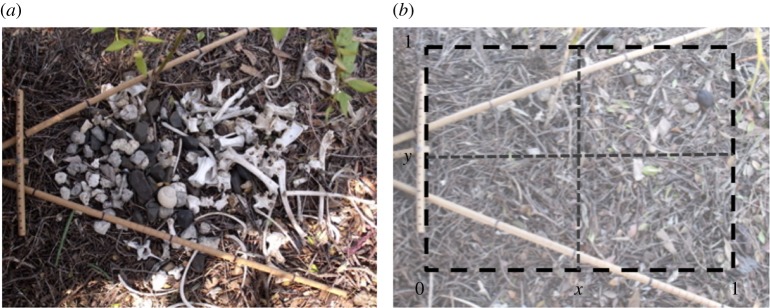
(*a*) Photograph of an undisturbed bower court, where the court entrance can be seen on the left-hand side. Wooden dowels mark the limits of the female field of view from within the bower. (*b*) Photograph of bower when all court objects have been removed, with overlaid schematic of court measurements used when objects were replaced.

Male bowerbirds (family Ptilonorhynchidae) build and decorate structures called bowers that are used in mate choice [9]. Great bowerbird (*Ptilonorhynchus nuchalis*) males build bowers comprising two parallel densely thatched stick walls and floor that create an avenue of up to 1 m long. Each end of the avenue opens up onto a display court, where a cleared area of ground is covered with tens to hundreds of grey and white objects such as stones, shells and bones (figure 1). These objects provide a background against which the male presents coloured decorations during display [10]. The objects on the display court are not placed randomly, but are arranged by size so that smaller objects are closer to the avenue and larger objects are further away (i.e. in a positive size–distance gradient, hereafter referred to as ‘gradient’) [11]. When a female views the court from inside the avenue during the male's display, this arrangement forms an even mosaic of visual angles that creates a forced perspective illusion. This illusion is important in mate choice, as males that create high-quality illusions (i.e. smaller standard deviation (s.d.) in visual angles created by object location) can gain more mates than rivals [5].

Males show consistent, individual differences in their gradients, which may reflect some aspect of male ability to learn and/or remember object arrangement [12]. Objects do not have specific locations on the court and males seem to organize objects based on their general size [11]. The consistency of object arrangement may be an important component of the signal given that females visit bowers multiple times before choosing a mate: in the closely related satin bowerbird (*P. violaceus*), females sample several males multiple times before choosing who to mate with each year [13] and will return to attractive males in subsequent years [14]. Being able to produce a consistent signal may be an indicator of a male's ability to maintain that signal at a fixed level that females can use to assess male quality within and across years, alongside the quality of the signal itself.

Object availability may also be an important factor determining gradient characteristics: males given objects from another bower created gradients similar to the donor bower rather than their original gradient [15]. However, there is no spatial relationship between bower location and gradient quality [12], so the variety of object sizes required to construct a high-quality gradient may also reflect superior male object foraging ability. Moreover, when gradients were improved or made worse by rearranging objects within courts, birds restored their original gradients within 3 days [11,12], suggesting that object availability is insufficient by itself to explain individual variation in gradient quality.

**Figure 2.**
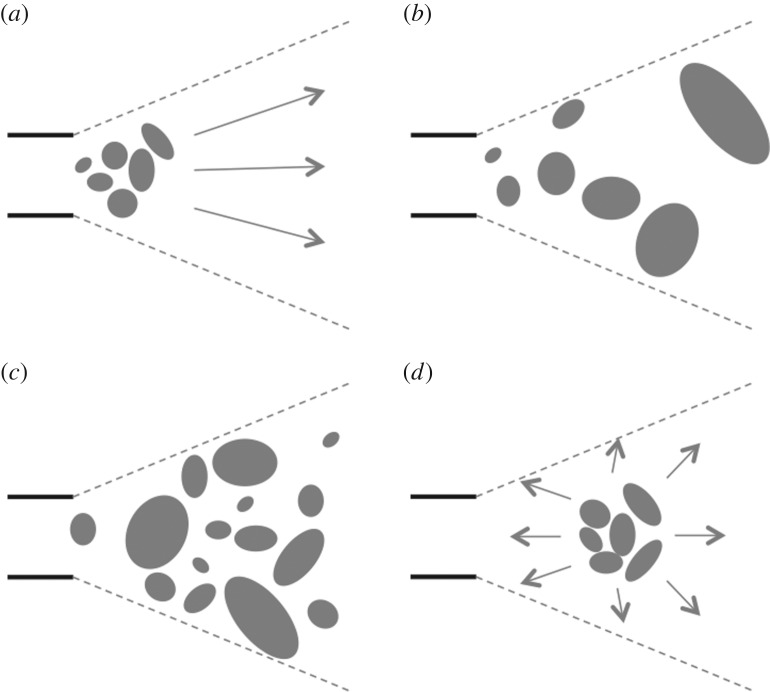
Simplified diagram showing potential reconstruction styles, horizontal parallel lines represent the bower entrance and grey dotted lines represent the female field of view. (*a*) Smallest objects closest into the bower and then increasing object size as construction progresses outwards (or vice versa); (*b*) random selection and placement of the correct sized objects in the appropriate court area in no particular order; (*c*) placement of all objects back on the court and then rearrangement by size; (*d*) placement of correct-sized objects in the centre of the court and then working outwards.

It is difficult to determine the extent of a cognitive component in gradient construction without first knowing how gradients are built, whether building behaviour varies among males, and whether variation in building behaviour is related to male quality. To address these questions, we moved the objects off one court of each bower of 14 male bowerbirds, and recorded how they reconstructed their gradients. Males could reconstruct their gradients in a number of ways: (i) by placing the smallest objects closest into the bower and then increasing object size as they moved further out from the bower avenue (or vice versa, starting with largest objects and working inwards towards the avenue); (ii) by randomly selecting and placing the correct sized objects in the appropriate court area in no particular order; (iii) by placing all objects back on the court and then rearranging them by size or (iv) by placing objects in the centre of the court and then working outwards, a technique commonly used by humans in building mosaics [16] (figure 2). Reconstruction strategy may also affect when the conditions for the illusion are recreated, for example, if all objects are placed randomly on the court and then arranged into a size–distance gradient, the illusion will not be present when the first objects are placed. However, if objects are ‘correctly’ placed according to size, then the conditions for the illusion will be present from the first few objects placed. Furthermore, illusion quality may remain stable or fluctuate as new objects are added, which may reflect individual skill.

We quantified how males reconstructed their gradients (and therefore the visual illusion) by measuring the location and time of each object placed and the changes in gradient over time. We also determined whether the first ten objects placed recreated a positive size–distance gradient and compared how the reconstructed gradient compared with the original gradient.

## 2. Material and methods

This study was carried out at Dreghorn Station in Queensland, Australia (20.25° S, 147.73° E). At the start of the breeding season in September 2011, motion-activated, solar-powered cameras were set up at 14 male great bowerbird bowers as described previously [5]. One camera was placed approximately 1 m away from the bower and was focused on the primary display court and aimed down the main avenue axis. The primary display court was identified as the court that had the most decorations, and is typically used most often during displays. A secondary camera was attached to a shrub branch above this court to provide a top-down view of the court. The cameras were set to record during daylight hours (05.30 to 18.00 h).

To quantify the quality of male courts during the breeding season, photographs of both courts were taken approximately every 8 days as described previously [11]. Wooden dowels with marks at 1 cm increments were used to mark out the female's maximum field of view of the entire court from within the avenue, and a *t*-shaped dowel set was used to locate and mark the centre of the entrance to the avenue and the distance to the female's location in the central depression of the avenue during male displays (figure 1*a*). The overhead camera was also calibrated with the dowels (to account for differences among bowers in the distance of the camera from the court), so that video frames could be exported and court objects measured in the same way as the photographs.

Court measurements were made using photographs and exported video frames using Matlab [11]. Court geometry was assessed using three measures: slope, visual angle variation and effect size [5,11]. The slope of each gradient on each court was calculated by regressing the visible width and visible depth of each court object against distance from the female's viewing location in the middle of the avenue. We calculated the visual angle that every court object subtended onto the female's eye when standing in the avenue. The regularity of the pattern is measured by the s.d. of visual angles; a smaller s.d. is associated with a more regular pattern. The effect size was calculated by comparing the actual perspective quality with 20 000 permutations (10 000 permutations estimates the *p*-value with a precision of 0.005) of what the bird could make with the objects available on the court [11].

Prior to manipulation, we photographed both courts at each bower. We then moved all of the objects (both coloured and uncoloured) off the primary display court and placed the objects 20 cm to one side of the edge of the court. After manipulation, each bower was left undisturbed for 72 h, and then another set of photographs of both bower courts was taken. The calibrated motion-activated cameras were on continuously during these times.

To determine how males began reconstruction of their courts, for each bower we extracted time-stamped video frames every time a new object was placed onto the court for the first 10 objects placed. We also determined where males placed the first 10 objects in relation to the distance from the avenue entrance and distance from the avenue long axis. In order to compare bowers with different court sizes, we assessed object placement relative to the original court length and width. During placement of the first 10 objects, dappled shade on four bowers made the object boundaries difficult to distinguish and data from these four males were excluded from further analysis. This left us with first 10 object placement data for 10 bowers.

We tested whether males reconstructed the original gradient values immediately by measuring how the gradient created by the first few objects compared with the original gradient. We did this for the fourth to tenth objects added to the court, when there were enough objects present to create a statistically reasonable size–distance gradient. Each time a new object was added to the court, we calculated the residual of each object from the original gradient residual, then calculated the root mean square (RMS) of these residuals; the s.d. of the residuals. If the birds had an inherent sense of perspective, we predicted that all objects placed would have similar RMS residuals to the original gradient, whereas trial-and-error placement would have more variable RMS residuals.

We also carried out overall comparisons of geometric quality calculating the residuals of the gradient regressions and the residuals from the mean visual angle (this is the s.d. of the visual angles). The smaller the residuals from the regression, or the smaller the visual angle s.d., the higher the geometric quality. We calculated three different sets of residuals from both the gradients and s.d. angles. R1 is the set of residuals of original objects from the original (pre-removal) value. It is a measure of the quality of the original court geometry. R2 is the set of residuals of the first 10 objects from their own gradient or s.d. angles. It is a measure of how carefully the male placed the first 10 objects in relation to each other; small R2 indicates a high-quality gradient or low s.d. with only 10 objects. R3 is the set of residuals of the first 10 objects from the original (pre-removal) value. It is a measure of how well males recovered their original gradient when placing the first 10 objects; small R3 indicates a good fit with the original gradient. We then compared R1 with R2 and R3 separately for each bower and separately for visible width and depth measurements. Probabilities of these differences were calculated for each bower using 20 000 permutations of the individual object residuals between the two groups being compared. If males were selecting and placing objects at random with respect to size and position, then we would expect the R2–R1 and R3–R1 differences to be significantly larger than zero. If males were placing objects non-randomly and consistently, we expected these differences to be not significantly different from zero. We also investigated whether motivation to replace the gradient was linked with mating success (the number of copulations recorded within the bower avenue), by determining whether the number of objects present on the court was associated with mating success.

To quantify how courts were reconstructed, we measured the court variables at 2 h intervals during daylight hours for 3 days after the courts were cleared (*n* = 14). Two bowers were excluded from analysis: one where the individual was highly atypical and had only a handful of objects on the court and another where the dappled lighting conditions made it difficult to accurately measure court objects, leaving 12 bowers. Court statistics as described previously were calculated at 2 h intervals during daylight to determine how the size–distance gradient, visual angle s.d. and effect size changed over 3 days. These data were analysed using generalized additive mixed models (GAMM) in the R gamm4 package [17,18] due to the nonlinear change of measures with time. We ran separate models for each response variable (slope of visible width, slope of visible depth, width visual angle s.d., depth visual angle s.d., width effect size and depth effect size), with time and number of objects as fixed effects and bower owner as a random effect. We then used the AIC values to select the most appropriate models.

## 3. Results

**Figure 3.**
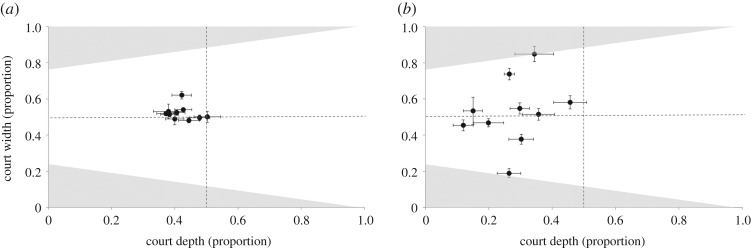
Relative locations of objects on the courts, each of the 10 points represent a bower and lines indicate the mean ± s.e. The dotted line *y* = 0.5 represents the female's central line of sight from the inside of the avenue, and the line at *x* = 0.5 represents the middle of the court's *x*-axis. Shaded grey areas are outside the female's field of view. (*a*) Locations of all objects on the court prior to removal and (*b*) locations of the first 10 objects placed on the court.

**Figure 4.**
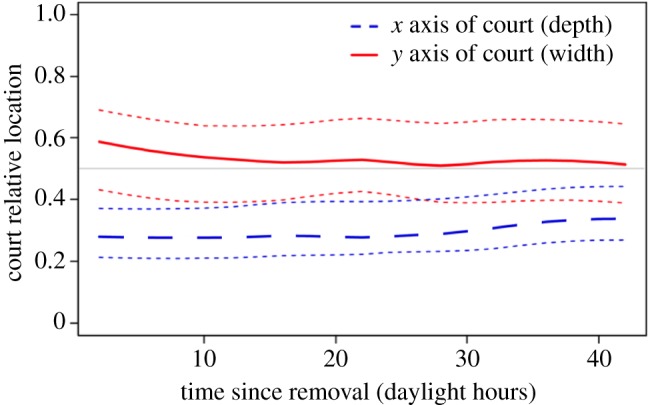
Average location of objects placed on courts measured every 2 h during daylight. The dashed line in blue indicates the *x*-axis (along avenue axis position) mean (dotted lines are ±s.e.). The continuous line in red indicates the *y*-axis (left–right) mean (±s.e.).

All males began reconstruction of their court gradients within 4 h of removal, with the first object being placed at an average time of 45 ± 17 min after court clearance (mean ± s.e., *n* = 12). On the *x*-axis of the court (along the avenue axis), all males placed the first 10 objects in the half of the court closest to the avenue compared with the original court, and 70% were in the closest third of the court (mean position = 0.25 ± 0.03; *n* = 10, figure 3). On the *y*-axis (distance to left or right of the avenue axis), seven of the 10 males placed their first 10 objects in the middle third of the court (i.e. where they were directly in the female's line of sight; mean position = 0.53 ± 0.06, centre at 0.5). Over the course of 72 h, males continued to place objects in the same pattern as seen in the placement of the first 10 objects—directly in the female's eyeline (court width), and in the first third of the court *x*-axis (figure 4). Males gradually increased the distance from the bower over time on the *x*-axis, with the average location of objects being 7% further away from the bower after 72 h (figure 4). The average location of objects on the *y*-axis moved 3% to the right after 72 h. After 72 h males had replaced 52 ± 6% of the original court objects. The s.d. in size of the original 10 objects was not significantly different from the s.d. of objects placed later on (Wilcoxon's test, all *p* > 0.5).

**Figure 5.**
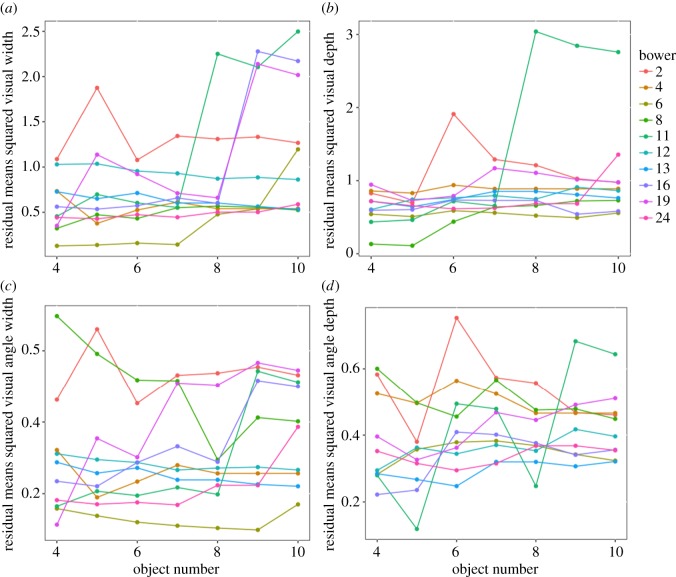
Measures (residuals) of how court measurements change as objects 4 to 10 are added: (*a*) visual width, (*b*) visual depth, (*c*) s.d. visual width angle and (*d*) s.d. visual depth angle. Lines connect values within bowers. The *y*-axis represents how similar court values are to original, pre-removal value; larger values indicate bigger differences.

Males differed in how well they recovered their gradients and visual angle evenness when placing the first 10 objects (figure 5). Most males had low values and low variation in their residuals, showing that they recreated and maintained the court values to their original individual level when adding the first objects. We compared residuals in order to assess the resemblance between the results of the first 10 objects and the pre-removal court geometry, where residuals R1 is a measure of the quality of the original court geometry, R2 is a measure of how careful the male was during placement of the first 10 objects and R3 is a measure of how well males recovered their original gradient. Two gradient comparisons were made by means of permutation tests in each bower: (i) between R1 and R2, and (ii) between R1 and R3. Two similar permutation tests were also made using the visual angle s.d.. Comparison (i) compares the quality of the geometry before removal and after the male put the first 10 objects back, and (ii) assesses how similar the recovered gradient was to the original. Most tests were non-significant; the mean probabilities were 0.084 (R1–R2 width), 0.12 (R1–R3 width), 0.21 (R1–R2 width angles), 0.22 (R1–R3 width angles), 0.16 (R1–R2 depth), 0.21 (R1–R3 depth), 0.22 (R1–R2 depth angle) and 0.22 (R1–R3 depth angle); see electronic supplementary material, table S1. After a sequential Bonferroni correction only two out of the 80 tests were significant with the first 10 residuals smaller than the original, and both of these showed negative differences, i.e. the first 10 objects placed created gradients that were better than the original gradients. Although the average differences in gradients were negative (see electronic supplementary material, table S1), none of the sign tests comparing positive and negative differences were significant (all *p *> 0.38). In summary, within bowers the gradients and resulting visual angles of the courts with the first 10 objects were similar to the pre-removal statistics and had similar gradient qualities. The number of objects present on the court was not correlated with court quality after 72 h (Pearson's correlation: object number versus 72 h RMS, all *p* > 0.05), or mating success (Spearman's rank: object number versus number of matings *r* = 0.35, *p* = 0.32).

**Figure 6.**
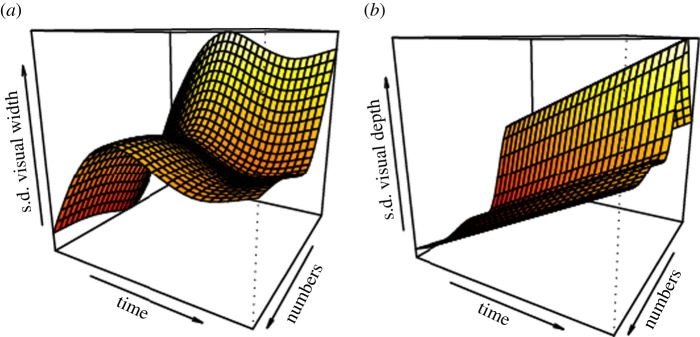
GAMM showing change in s.d. visual width (*a*) and visual depth (*b*) as a function of object numbers and time, accounting for bower identity. Visual angle s.d. width increased (i.e. quality decreased) and then became stable as time passed, whereas this measure decreased rapidly at first as object number increased (i.e. quality increased), and then remained stable. Visual angle s.d. depth increased over time. As object numbers increased the depth s.d. initially increased, then decreased and remained stable.

We then investigated how 72 h of reconstruction time and object number affected court measurements once variation across bowers was accounted for. The visible width gradient increased over time but the number of objects did not affect the slope, whereas the slope of visible depth was not affected by time or the number of objects used (table 1). Visual angle s.d. width and depth did not change consistently with increasing time or number of objects; court quality increased and decreased at various stages of court reconstruction (figure 6). Effect size measures how well the males create the illusion given the objects that are present on the court. As males increased the number of objects they had on the courts, the effect size increased for measures of width and depth, i.e. more objects allowed them to create higher quality gradients (table 1).

**Table 1.** GAMM results showing effects of time and number of objects on the court upon different gradient measures.

model and terms	d.f.	*F*	*p-*value	*r^2^*
slope width				0.02
time	2.62	3.43	0.02	
slope depth				0.02
time	1.00	1.71	0.19	
numbers	2.61	0.93	0.54	
s.d. visual angle width				0.05
time	3.03	3.74	0.01	
numbers	2.47	2.33	0.12	
s.d. visual angle depth				0.11
time	1.00	8.67	0.004	
numbers	4.93	3.66	0.005	
effect size width				0.44
numbers	1.43	113.6	>0.001	
effect size depth				0.20
numbers	6.50	8.14	>0.001	

## 4. Discussion

Males were motivated to replace their courts as they began reconstruction of their size–distance gradients within 45 min. They first replaced the section of the court that is most central in the female's field of view from within the bower—the middle of the court's *y*-axis and the half of the court closest to the bower entrance on the *x*-axis. The central court area is probably replaced first because it creates the backdrop for the male's courtship display that the female is guaranteed to see from her position in the bower [10]. Females appear to attend more to pattern regularity in visual depth than width when assessing courts during mate choice [5], which may explain why males replaced a greater area of the visual depth component compared with visual width. Replacing objects in the centre first may also be the simplest way to organize objects when placement location is important and space is constrained; this technique is also commonly used in human art when building mosaics [16], which are structurally similar to bowerbird courts.

We found that the depth and width components of the forced perspective illusion did not show a steady improvement during the time taken for the first 10 objects to be replaced. This suggests that there are complex relationships between the number and size distribution of objects, the time taken to place the objects and visual angles. This may reflect the shape of many objects on the court; any object that is not circular will show a simultaneous increase in visual depth and a decrease in visual width (or vice versa) when rotated [19]. Males must optimize the orientation of non-circular objects as well as their size and distance choices, so the lack of consistent improvement in illusion quality may reflect these challenges.

All males placed the first 10 objects in the same area of the court, indicating that they selected objects of a specific size to replace onto the court first and were not simply taking objects that were at the top of the pile; i.e. they actively searched for objects of the appropriate size to place in the centre area of the court. The ability to select appropriately sized objects for a task (without trial and error) is also seen in corvid tool use and zebra finch (*Taeniopygia guttata*) nest building [20,21]. However, male bowerbirds re-use approximately 38 ± 12% of objects from the previous year when they reconstruct their bower each year [22], and so may become familiar with some of the object size range they have available for construction. When males were given a new set of court objects from another bower they did not create gradients with the same characteristics (slope width/depth and s.d. visual angle width/depth) as their original court, which may be a result of completely unfamiliar object types and sizes [15]. However, one limitation of that study was a lack of size variation in transplanted objects, as court objects were exchanged between a sub-population of bowers with large variation in the size of court objects and a sub-population with court objects of roughly the same size (snail shells). The bowers that received transplanted objects with low size variance were, therefore, limited in the quality of gradient that they could construct using those objects.

Removing all objects from the courts and giving all males the same set of objects could determine the effect of object familiarity. If familiarity is important, we would expect that all males would create new illusions of a lower quality compared with their original illusion. If familiarity is unimportant, it seems likely that males learn to select appropriately sized objects for the centre of the court when they learn how to build courts. In a follow-up study, we swapped court objects between bowers within sub-populations that had similar variance in object sizes. We found that males created courts with gradient/visual angles strongly correlated with the one they had previously (A Rodrigues & JA Endler 2014, unpublished data). Object familiarity, therefore, appears to be of limited importance during court construction, and we predict that variation in the environment and object availability would select for males to be flexible in terms of the objects used and their placement given the largest and smallest objects available.

The speed of court and gradient repair indicates that males were motivated to replace the missing component of their courtship display. This is consistent with previous findings in this and other bowerbird species [11,12,23]. It could also be an indicator of male quality or experience. We showed previously that high-quality males (those with high-quality illusions) replaced a larger proportion of their original gradients within three days compared with lower quality males when gradients were improved [12]. However, this could be because moving a small number of objects would recreate the original gradient in a bower of high quality more easily compared with one of lower quality, rather than any indication of male skill. In this experiment, we found no relationship between the number of objects on the court after 72 h and (i) mating success or (ii) the quality of the court after 3 days. It is not the absolute number of objects that is important in creating a high-quality gradient, but the size distribution and placement of those objects, and high-quality males were more adept at doing this. After 3 days males had replaced approximately 50% of objects on their court, so although court reconstruction began rapidly and the visual conditions required for the illusion were present almost immediately, full gradient replacement took longer than 3 days.

Males did not appear to use trial and error when replacing their court objects. If males constructed gradients by trial and error, we would expect that the residuals of the first 10 objects should be much higher than the original geometry, and the locations to be random, but we found that the first 10 objects placed created court geometry that was the same as prior to removal. In fact, on average the quality of geometry created by the first 10 objects was slightly higher than the original, the opposite of what we expected from trial and error. This does not exclude the possibility that males used trial and error the first time they ever constructed the court geometry and then later recalled the general location of objects of certain sizes (a simple ‘rule of thumb’, i.e. knowledge of the approximate location of objects by size based on prior experience). Although previous work has shown that males do not place objects in exactly the same location as previously when the court is disturbed [11], a simple, general rule concerning location and object size could be used to create size–distance gradients.

Alternatively, males may have an internal template that can also be refined through learning (similar to song learning; [24]), either when they are immature and observing mature males building bowers, or when they first begin building their own bowers. There are two different possible templates: a template for the gradient itself and a template for the degree of evenness of the pattern as seen from inside the avenue. Discriminating between these potential explanations is difficult because although the underlying cognitive processes involved may differ, the behavioural outcomes may be indistinguishable. The construction behaviour of individually identifiable immature males must be compared with that of their ‘tutor’ male and also quantified from the first year of bower building and over consecutive years to further investigate how gradients are constructed. If males have some form of internal template then objects should be immediately placed in the correct position, whereas if using ‘rule of thumb’ objects may be rearranged more. Identifying changes in construction behaviour over time can also address the relative contributions of social learning from a tutor, when an immature male observes a mature male building his gradient, and solitary learning, when a male learns to build his own gradient.

We expect that energetically or cognitively demanding building behaviour will be a feature of structures that are used in mate choice, as they can provide information about male quality to females [4]. Male black wheatears (*Oenanthe leucura*), for example, carry stones to their nest and females use the number of stones carried (rather than the actual number of stones present at the nest) to assess male quality [25]. Wheatear male quality is assessed based on the energy requirements involved in creating the ornament, rather than the ornament itself. The craters created by bower building cichlids are also likely to be energetically costly to build, maintain and defend, as males with larger bowers gain more mates [26,27]. In this case, female choice is likely to be based on a simple measure of size rather than some specific detail of the ornament. In animals that create more complex constructions, such as great bowerbird forced perspective illusions and perhaps the recently described geometric circles created by male pufferfish (*Torquigener* sp.), the finer details of the construction are assessed [5,8]. The potential cognitive challenges involved in building similar structures, and the information that they convey about male quality, invites further exploration.

## Supplementary Material

Mean differences between sets of residuals and permutation test probabilities of the differences

## Data accessibility

Data are available at https://dx.doi.org/10.6084/m9.figshare.4138602.v1.
